# Peroxiredoxin 6 in Stress Orchestration and Disease Interplay

**DOI:** 10.3390/antiox14040379

**Published:** 2025-03-23

**Authors:** Jiangfeng Liao, Yusi Zhang, Jianwei Yang, Longfei Chen, Jing Zhang, Xiaochun Chen

**Affiliations:** 1 Department of Neurology, Institute of Neurology, The First Affiliated Hospital of Fujian Medical University, Fuzhou 350001, China; jf.liao@fjmu.edu.cn (J.L.); yangjianwei@fjmu.edu.cn (J.Y.); clffjfz@fjmu.edu.cn (L.C.); 2 Department of Neurology, National Regional Medical Center, Binhai Campus of the First Affiliated Hospital, Fujian Medical University, Fuzhou 350212, China; 3 Institute of Neuroscience, Fujian Key Laboratory of Molecular Neurology, Fujian Medical University, Fuzhou 350004, China; fyxhzys@fjmu.edu.cn

**Keywords:** peroxiredoxin 6, various stressors, structure-based functional switches, PRDX6-related disorders, exogenous supplementation

## Abstract

As a moonlighting protein with multiple enzymatic activities, peroxiredoxin 6 (PRDX6) maintains redox homeostasis, regulates phospholipid metabolism, and mediates intra- and inter-cellular signaling transduction. Its expression and activity can be regulated by diverse stressors. However, the roles and relevant mechanisms of these regulators in various conditions have yet to be comprehensively reviewed. In this study, these stressors were systematically reviewed both in vivo and in vitro and classified into chemical, physical, and biological categories. We found that the regulatory effects of these stressors on PRDX6 expression were primarily mediated via key transcriptional factors (e.g., NRF2, HIF-1α, SP1, and NF-κB), micro-RNAs, and receptor- or kinase-dependent signaling pathways. Additionally, certain stressors, including reactive oxygen species, pH fluctuations, and post-translational modifications, induced the structure-based functional switches in the PRDX6 enzyme. We further reviewed the altered expression of PRDX6 under various disease conditions, with a particular focus on neuropsychiatric disorders and cancers, and proposed the concept of PRDX6-related disorders (PRD), which refers to a spectrum of diseases mediated by or associated with dysregulated PRDX6 expression. Finally, we found that an exogenous supplementation of PRDX6 protein provided preventive and therapeutic potentials for oxidative stress-related injuries in both in vivo and in vitro models. Taken together, this review underscores the critical role of PRDX6 as a cellular orchestrator in response to various stressors, highlighting its clinical potential for disease monitoring and the development of therapeutic strategies.

## 1. Introduction

Peroxiredoxins (PRDXs) are members of an evolutionarily conserved family of peroxidases capable of reducing a wide range of peroxide substrates [1,2]. These enzymes are ubiquitously expressed across all major organs of mammals and can be classified into three categories based on the number of the residues of conserved cysteines (Cys) involved in catalysis: typical 2-Cystein PRDXs (PRDX1-4), atypical 2-Cystein PRDX5, and 1-Cystein PRDX6 [2]. All PRDX isoforms share a fundamental catalytic mechanism, where a reduced Cys (-SH) at the active site is oxidized into a sulfenic form (-SOH) by peroxide substrates. However, they differ greatly in their abilities to recycle sulfenic acid back to thiol forms and in restoring hyperoxidized states, such as sulfinic acid (-SO_2_H) and sulfonic acid (-SO_3_H) [3]. It has been reported that the oxidized sulfenic form of PRDX6 can be reduced, whereas its hyperoxidized sulfinic and sulfonic forms are not reversible, contrary to the hyperoxidized sulfinic forms of other PRDXs that can be restored through the action of ATP-dependent enzyme sulfiredoxin [4]. Therefore, PRDXs play a crucial role in regulating cellular redox homeostasis and oxidative stress responses.

PRDX6 is a particularly intriguing member of the PRDXs. Ever since its initial cloning from mouse kidneys in 1997, it has received growing attention in various research fields [5]. Over the past two decades, constant efforts have been dedicated to elucidating its pathophysiological implications in living organisms. PRDX6 is widely recognized as a moonlighting protein with multiple enzymatic activities, including glutathinone peroxidase (Gpx), acidic calcium-independent phospholipase (aiPLA2), and lysophosphatidylcholine acyl transferase (LPCAT) [6], with its enzymatic functions varying under distinct conditions [1]. For instance, the optimal pH for the activities of Gpx and aiPLA2 of PRDX6 varies considerably; Gpx activity maximizes at a pH exceeding 7 in the cytosol, while aiPLA2 activity peaks at pH 4 within lysosomal lamellar bodies. Notably, PRDX6 is unique not only for its ability to utilize both glutathione and phospholipid hydroperoxides as substrates but also for its crucial downstream cellular signaling effects [1,7]. Its enzymatic activities are closely associated with lipid peroxidation, selenocysteine metabolism and ferroptosis, and the activation of NADPH oxidases and redox-dependent inflammatory pathways [8,9]. Hence, the complex interplay of these enzymatic activities suggests that PRDX6 may serve as a crucial molecular node in various pathophysiological conditions.

Emerging evidence has intriguingly indicated that the expression and functional dynamics of PRDX6 are substantially modulated by various endogenous and exogenous stressors [2,10], making this area a subject of considerable interest. Given the complex and sometimes contradictory results, a comprehensive overview of these regulators is essential to enhance our understanding of this enzyme and its potential applications. The current study systematically delineated the spectrum of in vivo and in vitro stressors that modulate PRDX6 expression and subsequently examined its structural and functional adaptations, relevant pathological implications, and therapeutic prospects in exogenous supplementation. Based on the current literature, this review highlights PRDX6 as a crucial orchestrator against various internal and external stressors, signifying its potential applications in diagnostic and therapeutic strategies for stress-related disorders.

## 2. Dysregulation of PRDX6 Expression Induced by Various Stressors

Previous web-based computer analyses of the 5′-flanking regions of the PRDX6 gene promoter have identified multiple putative regulatory elements, including binding sites for specificity protein 1 (SP1), nuclear factor erythroid 2-related factor 2 (NRF2), and hypoxia-inducible factor 1 alpha (HIF-1α) [11]. The available evidence suggests that a variety of stressors can modulate PRDX6 expression both in vivo and in vitro through transcriptional factor binding, rendering it an important responder to both internal disruption and external insults. These stressors may be roughly classified into chemical, physical, and biological types. In some cases, the expression of PRDX6 may exhibit a biphasic response pattern depending on both the duration and intensity of stressor exposure. Therefore, a further summary of their regulations on PRDX6 expression and the underlying mechanisms will significantly enhance our understanding of its biological functions and molecular features.

### 2.1. In Vivo Dysregulation of PRDX6 Expression Induced by Various Stressors

Previous studies have revealed that various chemical compounds can induce the dysregulation of PRDX6 expression. For instance, magnesium sulfate (MgSO_4_) has been shown to provide protection against oxidative damage and inflammatory response in the rat placenta within a model of intrahepatic cholestasis of pregnancy by enhancing the NRF2/PRDX6 signaling pathway [12]. The treatment with cobalt chloride (CoCl_2_) has been found to increase the expressions of two PRDX6-related redox-active transcription factors (NRF2 and HIF-1α) to ameliorate oxidative stress in auditory cells [13]. In addition, several sulfur agents, such as sulfur mustard, hydrogen sulfide, and buthionine sulfoximine, may also exert antioxidant effects by elevating the PRDX6 expression via their transcriptional effects [14,15,16]. In contrast, the expression of PRDX6 is downregulated in the livers of ethanol-fed rodents, accompanied by the activation of nuclear factor kappa B (NF-κB) and phosphorylation of mitogen-activated protein kinases (MAPKs) [17,18], implying the critical roles of PRDX6 in attenuating cellular inflammation and oxidative stress.

Notably, carbon tetrachloride (CCl_4_) has been reported to inhibit hepatic fibrosis but appears to modulate the PRDX6 expression bidirectionally [19,20]. Specifically, both the protein and mRNA levels of PRDX6 are elicited in hepatic fibrosis induced by CCl_4_ after four weeks; however, eight weeks after the treatment, only the mRNA level of PRDX6 is increased while its protein level shows a comparative decrease. This paradox may be attributed to the release of mesencephalic astrocyte-derived neurotrophic factor (MANF), which promotes the PRDX6 release in relation to the intensity and duration of CCl_4_ exposure or to the protein leakage from apoptotic or necrotic hepatocytes caused by CCl_4_ [19]. Collectively, these findings underscore the complex regulatory effects that chemical agents have on PRDX6 expression and highlight the critical roles played by various transcriptional factors (e.g., NRF2 and NF-κB) in modulating its expression.

PRDX6 has also been shown to underlie the effects of several chemical stressors on male infertility. For example, an exposure to 4-tert-octylphenol or paclitaxel can impair sperm motility and viability by downregulating the PRDX6 levels, alongside the inhibition of the activities of protein kinase A/C (PKA/C), sirtuin 1 (SIRT1), or NRF2 [21,22]. On the other hand, a controlled clinical trial reports that the expression of semen PRDX6 is significantly elevated following the consumption of date palm pollen, which positively correlates with improved parameters of sperm, including count, volume, motility, and morphology [23]. Rodent studies have also demonstrated a marked upregulation of protective PRDX6 protein in both the epididymis and spermatozoa upon exposure to Di-n-butyl phthalate or tert-butyl hydroperoxide [24,25]. Although the precise molecular mechanisms underlying these observations remain largely elusive, these findings have established a strong link between PRDX6 dysregulation and male reproductive health, indicating the critical role of this antioxidant enzyme in the pathogenesis of male infertility.

Apart from the various chemical factors mentioned above, the alteration in PRDX6 expression can be influenced by certain physical stressors. Previous proteomic analyses have identified an upregulation of PRDX6 in both the spleen and liver of mice when exposed to proton beam (2 Gy), ionizing radiation (10 Gy), and carbon nanotubes [26,27,28]; while an exposure to electron beam irradiation (45 Gy) or ultraviolet-B can downregulate the expression of PRDX6 in rodent skin by regulating miR-214 and BMAL1 (brain and muscle ARNT-like protein 1) signals [29,30]. Other physical factors, such as temperature [31,32], hyperoxia [33], or hypoxia [32], have also been documented to impact the expression of PRDX6. These findings indicate the significant associations between the alteration in PRDX6 expression and cellular antioxidant defense mechanisms.

Additionally, physiological activities, such as exercise and reproductive activities (e.g., mating or fertilization), have been shown to stimulate the expression of PRDX6 in human blood [34] and rodent oviducts or colons [35,36]. Biological infections caused by bacterial, viral, or parasitic agents can disrupt immune homeostasis in mouse pulmonary and hepatic systems by affecting PRDX6 expression possibly through several transcriptional factors, such as NF-κB, NRF2, and CCAAT/enhancer-binding protein beta (C/EBPβ) [37,38,39].

A comprehensive summary of additional external stimuli contributing to PRDX6 dysregulation in vivo is detailed in Table 1.

### 2.2. In Vitro Dysregulation of PRDX6 Expression Induced by Various Stressors

A growing body of research utilizing multiple cell lines has demonstrated the regulation of PRDX6 by various external stressors, several of which are particularly interesting and merit in-depth discussion. For instance, physical factors, such as ionizing radiation (2 or 20 Gy) and electromagnetic waves, can elevate the PRDX6 expression in both human epithelial cells and monocytes to maintain redox homeostasis and protect against oxidative stress and inflammation [70,71,72]. More intriguingly, certain biochemical agents, including curcumin, hydrogen peroxide (H_2_O_2_), and inflammatory cytokines, have been extensively investigated for their distinct regulatory effects on the PRDX6 expression [73,74] and will be overarchingly summarized below.

As a polyphenol extracted from turmeric rhizome, the mechanism underlying the protective effects of curcumin has been a research focus for decades. Treatment with curcumin, at doses of 5 µM for 48 h or 109 µM for 2 h, has been reported to mitigate inflammation and oxidative stress by increasing PRDX6 expression possibly through the enhanced transcriptional activities of specificity protein 1 (SP1) [74,75]. On the other hand, curcumin administered at a dose of 1 µM for 24 h decreases the PRDX6 level to inhibit astrocyte activation [76]. Proteomics analyses have also indicated that the administration of curcumin at doses ranging from 25 µM or 1737 µM over a period of 72 h can reverse multi-drug resistance in tumors and inhibit tumor cell proliferation by reducing the PRDX6 levels through p53 [77,78]. Therefore, it is reasonable to conclude that PRDX6 serves as a crucial downstream target in curcumin-conferred protections, although there is still no consensus regarding its optimal dosage and timing on protein expression. In addition to curcumin, an array of phytochemicals, such as baicalein, luteolin, andrographolide, and glycyrrhizin, have been extensively documented as potent modulators for PRDX6 expression [79,80,81,82,83].

Another intriguing example is H_2_O_2_, a well-known inducer of oxidative stress, which forms a much more complex relation with PRDX6 expression. Specifically, previous studies have reported that H_2_O_2_ administered at a low dose (25 µM for 9 h) may increase the expression of PRDX6 in mouse HEI-OC1 cell line [13]. Moderate doses of H_2_O_2_ (e.g., 100 µM for 30 min, 1 h, or 24 h; and 300–500 µM for 6 h or 48 h) can reduce the cellular expression of PRDX6, accompanied by increased cellular apoptosis and oxidative stress [13,50,79,84,85,86]. However, an exposure to higher concentrations of H_2_O_2_ (1000 µM) may increase the PRDX6 levels in mouse H2.35 cells after treatment for either 8 h or 24 h [87], but decrease its expression in human APRE-19 cells after a duration of 48 h [84], suggesting potential dual effects of high concentration of H_2_O_2_ on the PRDX6 expression. These reports indicate that the H_2_O_2_-mediated regulation of PRDX6 is largely contingent upon the specific dosage and exposure time. Recent, more direct evidence shows that the gradient concentration of H_2_O_2_ (ranging from 50 to 200 µM over a period of 24 h and 50 µM across time points from 2 h to 24 h) gradually upregulates the PRDX6 levels in human astrocyte cell lines (HA-sp and A172) [76]. Collectively, H_2_O_2_ may exert influences on the expression of PRDX6 in a dose- and time-dependent manner, possibly associated with the extent of cellular oxidative stress.

Inflammatory cytokines have also been demonstrated to modulate the expression of PRDX6 bidirectionally. For instance, tumor necrosis factor alpha (TNFα) treatment has been shown to upregulate the PRDX6 level in human embryonic kidney cells during short exposure (5 h, 7 h, or 9 h), while downregulating its expression in rat pancreatic beta cells (RINm5F) and retinal ganglion cells following prolonged exposure (24 h, or 10 days). These alterations in PRDX6 expression are linked to critical cellular processes, including cell migration, oxidative vulnerability, and cell death [88,89,90]. Additionally, both interferon γ (140 U/mL for 24 h) and lipopolysaccharide (1, 10, or 20 µg/mL for 8 h or 24 h) can decrease the PRDX6 expression in insulin-producing RINm5F cells [89] and tracheobronchial epithelial cells [91], thereby enhancing cellular susceptibility to oxidative stress and suppressing inflammatory responses, respectively, while combinations of them in turn stimulate its expression in mouse bone-marrow-derived macrophages, conferring protection against oxidative and nitrosative stress [92]. These differences may, of course, be attributed to variations among the cell lines used, but when considering the intricate protein post-translational modifications and enzymatic activities of PRDX6, its expression can vary under different and even identical external stressors. Despite this, these results indicate PRDX6 as a key downstream molecular target of cellular inflammation or immune cascade. It is important to concurrently assess both the protein post-translational modification states and enzymatic activities of PRDX6 when detecting its expression to obtain a comprehensive landscape.

A more comprehensive summary of various external stimuli that influence the dysregulation of PRDX6 in vitro is detailed in Table 2.

Taken together, extensive evidence from vertebrate models to various cell lines has revealed that the expression of PRDX6 is susceptible to change by diverse external stressors, which can be systematically classified into three major categories: chemical, physical, and biological factors. Several mechanisms may account for their regulatory effects on PRDX6 expression: (1) modulation of the activities of key transcriptional factors, particularly NRF2, HIF-1α, SP1, and NF-κB; (2) specific inductions of micro-RNAs, notably miR-214 and miR-27b; (3) activation of receptor-mediated signaling pathways, such as glucagon-like peptide-1 receptor (GLP-1R), epidermal growth factor receptor (EGFR), and N-methyl-D-aspartate receptor (NMDAR); (4) protein kinase-dependent phosphorylation cascades, primarily through PKC and MAPKs; (5) direct binding to its gene response elements; and (6) additional mechanisms currently unidentified. This intricate regulatory network elucidates the sophisticated control of PRDX6 expression in response to various stressors, offering novel insights into cellular stress orchestration.

Nevertheless, the relation between PRDX6 expression and its functional ramifications remains poorly understood, primarily due to the lack of studies that have concurrently assessed changes in enzymatic activities. Of note, an elevated PRDX6 expression may fail to inherently confer a protective effect, as such a change may also indicate an adaptive context-dependent response that can be beneficial, neutral, or potentially detrimental. Furthermore, a significant proportion of proteomic studies have merely reported the changes in PRDX6 expression without elucidating their functional implications, thereby limiting comprehensive understanding of its biological roles. Collectively, these findings highlight the pivotal role of PRDX6 in response to external stressors, enhancing our understanding of its molecular characteristics and therapeutic potentials.

## 3. Conformational and Functional Switches of PRDX6 in Response to Stressors

**Figure 1:**
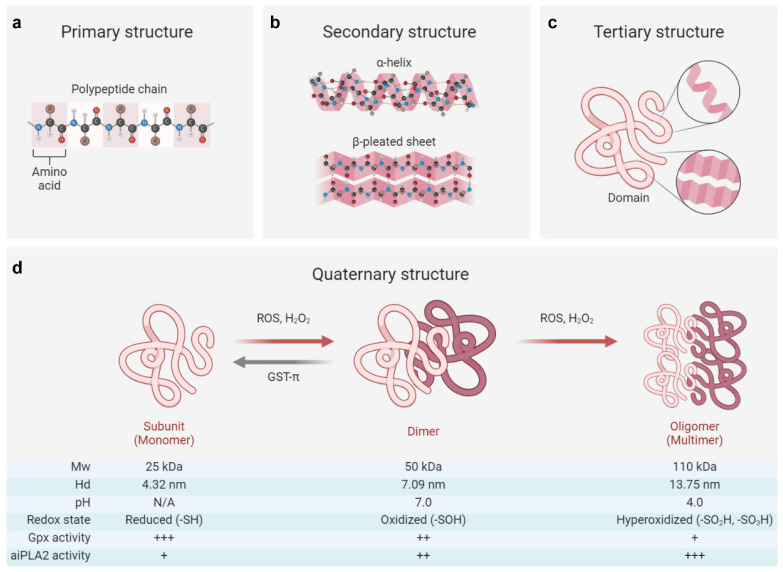
Structural and functional switch of the PRDX6 protein. (**a**–**c**) The primary structure of PRDX6 consists of a polypeptide chain composed of 224 amino acids, which subsequently folds into secondary structures (including α-helix and β-pleated sheet) and a tertiary domain [1,126,127]. (**d**) The quaternary structure of PRDX6 exhibits dynamic regulation in response to oxidative stress and pH variations, enabling its functional switching [3,128,129]. Briefly, the reduced form (-SH) of the PRDX6 monomer (Mw: ~25 kDa, Hd: ~4.32 nm) can transition to an oxidized form (-SOH) as a dimer (Mw: ~50 kDa, Hd: ~7.09 nm) in response to oxidative stress induced by reactive oxygen species (ROS) or hydrogen peroxide (H_2_O_2_). This transformation is often associated with attenuated Gpx activity and increased aiPLA2 activity, which can be reversed by enzymes such as glutathione S-transferase π (GST-π). On the other hand, the oxidized PRDX6 dimer may undergo further hyperoxidation (-SO_2_H; -SO_3_H), resulting in a multimeric oligomer form (Mw: ~110 kDa, Hd: ~13.75 nm), achieving its maximum aiPLA2 activity within acidic organelles at approximately pH 4. Of note, the oligomeric state of its reduced form (-SH) remains controversial, with conflicting reports suggesting either a monomeric [3] or dimeric [126,127,128] configuration in solution. Mw, molecular weight; Hd, hydrodynamic diameter; +, ++, and +++, respectively, indicating the relative intensity of enzyme activity. The figure was created with Biorender.com.

The primary structure of PRDX6 consists of a polypeptide chain comprising 224 amino acids, characterized by a single conserved redox-active C47 residue (peroxidative cysteine) at the N-terminal end of the α2 helix within βαβ motif of the thioredoxin fold. PRDX6 typically exists as a secondary or tertiary structure, forming a homodimer linked through hydrogen bonding networks between two β-strands from each monomer [126,127]. Under hyperoxidative conditions, PRDX6 may adopt an oligomeric quaternary structure [126]. Notably, both the internal and external stressors, such as reactive oxygen species, pH, and post-translational modifications, can regulate the conformation of PRDX6 [3,128,129], which is closely associated with its enzymatic properties (Figure 1).

Reactive oxygen species (ROS), particularly H_2_O_2_, function as critical messengers that regulate cellular activities through their interactions with the PRDX6 protein [73,130]. At low concentrations (<100 μM), H_2_O_2_ increases the PRDX6 expression via HIF-1α or NRF2 signaling pathways, while at higher concentrations (>100 μM), it can reduce the PRDX6 level by inhibiting the transcriptional activities of NRF2 and HIF-1α or activating the NF-κB signaling pathway [13,73,86]. Beyond regulating the expression of PRDX6, H_2_O_2_ also influences its structural adaptions [73]. Elevated concentrations of H_2_O_2_ (>100 μM) facilitate monomer–dimer transitions in PRDX6 and induce its transformation from dimeric to multimeric states [3,126]. Mass spectroscopy studies reveal that the monomeric form of PRDX6 (Mw: 25 kDa; Hd: 4.32 nm) exists in a reduced state while its dimeric counterpart (Mw: 50 kDa; Hd: 7.09 nm) is oxidized [3]. These oxidative modifications of PRDX6 profoundly impact its enzymatic functions and subsequent pathophysiological consequences.

Specifically, the oxidized (-SOH) form of PRDX6 can be reduced by glutathione S-transferase π (GST-π) in the presence of glutathione, providing a crucial compensatory mechanism against intracellular oxidative damage; in contrast, its hyperoxidized forms (-SO_2_H and -SO_3_H) are irreversible, exhibiting diminished Gpx activity but dramatically enhanced aiPLA2 activity [3,126]. The hyperoxidized PRDX6 has been implicated in exacerbating various pathological conditions, including acute lung injury, acute liver injury, and cerebral ischemia by triggering oxidative stress and inflammatory cascades [131,132,133]. Intriguingly, this hyperoxidized state may also confer antitumor effects by disrupting extracellular signal transduction pathways and inducing cell cycle arrest [4,126]. However, the precise relation between PRDX6 expression, the transitions of its oxidative state, and their pathophysiological consequences remains largely unknown and warrants further investigation.

Furthermore, it has been shown that the aiPLA2 activity of PRDX6 can be markedly augmented by MAPK-induced phosphorylation at the T177 residue [129]. This post-translational modification plays a crucial role in the degradation of dipalmitoyl phosphatidylcholine and the subsequent remodeling of phosphatidylcholines, thereby contributing to the pathogenesis of various diseases, such as drug addiction and acute lung injury [131,134]. Notably, the alteration in pH can also bring conformational changes in PRDX6, existing predominantly as a dimeric structure at physiological pH (7.0) while forming a tetrameric configuration (Mw: 110 kDa, Hd: 13.75 nm) under acidic conditions (pH 4.0). This structural plasticity provides a basis for its remarkable stability against lysosomal pH and thermal denaturation [128]. Additionally, pharmacological agents, such as acetaminophen and astragaloside IV, have been shown to modulate the conformational stability of PRDX6 through direct interactions with its catalytic sites, consequently impairing its antioxidant functions and cellular responses to oxidative stress [135,136].

Taken together, PRDX6 represents a structurally and functionally sophisticated enzyme whose quaternary structure is dynamically modulated by various stressors, with its multifaceted biological activities being intricately linked to its conformational states. Given the crucial role of such protein conformational and functional switches in physiological maintenance and pathological progression, further in-depth explorations are warranted to elucidate the molecular intricacies and pathophysiological implications of PRDX6 regulation.

## 4. The Close Involvement of PRDX6 in Diverse Pathological Conditions

PRDX6 has been implicated in the pathogenesis of various chronic noncommunicable diseases [1,2]. Our previous studies have particularly investigated its significant roles in the pathophysiology of the central nervous system, encompassing neurodegeneration, stroke, neurotrauma, gliomas, major depressive disorder, and post-traumatic stress disorder [1,137,138]. Recent advances in reproductive biology have identified PRDX6 as a key protector of spermatozoa against oxidative damage, primarily through its dual Gpx and aiPLA2 activities [8,139,140]. A deficiency in PRDX6 is associated with impaired sperm function and compromised DNA integrity in both human male infertility and rodent models [140,141], while its elevation may underlie the protective effects of curcumin and melatonin on sperm motility, viability, fertilization rates, and blastocyst development [42,54]. Notably, although the scientific community remains divided regarding its role in oncogenesis, compelling evidence suggests that PRDX6 exerts substantial influence on cancer initiation and progression across various malignancies [10], making it a focal point in the present review (Table 3).

The dysregulation of PRDX6 expression has been extensively documented across a spectrum of malignancies. Specifically, PRDX6 demonstrates tissue-specific expression patterns, with downregulation observed in renal cell carcinoma [142]; while a substantial body of pre-clinical and clinical studies suggests PRDX6 upregulation in melanoma [98] and various solid tumors, including brain tumors [1,143], lung cancer [142,144,145,146,147,148,149], cervical carcinoma [142,150], endometrial cancer [142,151], ovarian cancer [142], skin malignancies [152], head and neck squamous cell carcinoma [153], and bladder cancer [142,154,155]. A higher tissue content of PRDX6 is often correlated with lower overall survival rates and poorer outcomes [142,156,157,158], but paradox findings exist in certain cancer types. For instance, integrative bioinformatics analyses have suggested that increased expression of PRDX6 serves as a poor prognostic factor for liver cancers [142,159], while preclinical studies identify it as a suppressor for carcinogenesis in both liver and prostate tissues [160,161]. Similar results can be found in cancers from the colorectum [162,163,164], thyroid gland [142,165], and stomach [166,167]. Several plausible explanations may account for these discrepancies: (1) the complex enzymatic activities and extensive post-translational modifications of PRDX6 protein; (2) the substantial intrinsic heterogeneity across different cancer types; and (3) potential variations in experimental methodologies or differences among animal models employed in these studies.

Additionally, the enzymatic activity of PRDX6 typically correlates with its expression level; however, the predominant activities of PRDX6 enzymes can vary under specific pathological conditions. For instance, in the central nervous system, PRDX6-aiPLA2 has been reported to aggravate neuroinflammation following ischemic stroke by modulating astrocyte-induced M1 microglia activation and promoting the secretion of neurotoxic inflammatory mediators, including IL-1β, IL-17, and IL-23 [133,168]. Inhibition of its aiPLA2 activity significantly reduces neurologic deficits, cerebral infarction, brain water content and inflammatory molecules [168]. It has been observed that PRDX6 is upregulated in the hippocampus of chronic epilepsy rats, accompanied by an increase in aiPLA2 activity over Gpx activity in astrocytes, which leads to prolonged seizure activity due to the autophagic degeneration of astroglial cells [169]. Although PRDX6-aiPLA2 activity has been shown to promote cancer cell death induced by TNFα in hepatocellular carcinoma [160], previous research using specific pharmacologic inhibitors and mutagenesis studies has demonstrated the critical roles played by both enzyme activities for lung tumor development [170,171]. Specifically, PRDX6-Gpx activity facilitates lung cancer growth while its aiPLA2 activity enhances invasiveness. The latter is mediated by the accumulation of arachidonic acid and subsequent activation of invasive signaling pathways involving p38 MAPK, PI3K-Akt signaling cascades, and urokinase-type plasminogen activator [170]. Therefore, these findings underscore that both enzymatic activities of PRDX6 are closely implicated in disease onset and progression.

Emerging evidence has demonstrated the presence of PRDX6 in human body fluids. Specifically, the expression of PRDX6 is documented to elevate in the sera of patients with lung squamous cell carcinoma [147] or hepatocellular carcinoma [160], but decrease in both the sera of patients with esophageal or colon carcinoma [147] and the interstitial fluid of patients with breast cancer [158]. Comparative proteomics analysis has revealed that plasma-derived small extracellular vesicles containing a signature of PRDX6 are capable of predicting the response of locally advanced rectal cancer to neoadjuvant chemoradiotherapy [172]. In addition, PRDX6 also presents in human cerebral spinal fluid and urine [173,174]. These reports merely emphasize the significance of serum PRDX6 as a biomarker for cancer diagnosis and monitoring treatment efficacy, without due attention to the underlying mechanisms. Recent research has indicated that PRDX6 may be extracellularly secreted to eliminate the ROS via its aiPLA2 activity or membrane aquaporins [73,175]. It is noteworthy to mention that the presence of PRDX6 in blood may present as a tumor antigen. Indeed, to facilitate diagnosis of early-stage malignant tumors and novel effective immunotherapies, Nakanishi et al. have detected specific antibodies against PRDX6 in peripheral blood from patients with esophageal squamous carcinoma and lung adenocarcinoma [149,176]. Despite nearly two decades of effort, it remains unknown regarding the mechanisms underlying the generation of PRDX6 antibody and its functional implications in cancer pathogenesis. With advances in tumor immunology, further studies are warranted to elucidate the role of PRDX6 and its corresponding antibodies in tumorigenesis and cancer progression.

**Table 3:** The expressions and effects of PRDX6 across various solid cancers.

Cancer Types	Expressions		Effects	Samples	Methods	References
	Tissue	Fluid				
Glioma	↑	NA	+	H	2-DE, BA	[1,143]
Head and neck carcinoma	↑	NA	+	H	BA	[153]
Lung cancer	↑	↑ *	+	H, M, C	BA, qPCR, WB, IHC, ELISA, IF, 2-DE	[142,144,145,146,147,148,149]
Esophageal carcinoma	↑	↓ *	+	H, C	BA, WB, IHC, IF, 2-DE	[142,147,176,177]
Cervical cancer	↑	NA	+	H, M, C	BA, IHC, WB	[142,150]
Endometrial cancer	↑	NA	+	H, M, C	BA, WB	[142,151]
Ovarian cancer	↑	NA	+	H	BA	[142]
Melanoma	↑	NA	+	H, F, C	BA, qPCR, WB	[98,142]
Cholangiocarcinoma	↑	NA	+	H, R	IHC, IF, WB	[43]
Skin cancer	↑	NA	+/-	H, M	IHC	[152]
Bladder cancer	↑	NA	+/-	H	BA, IHC, qPCR	[142,154,155]
Breast carcinoma	↑/↓	↓ ^#^	+	H, M, C	2-DE, IHC, qPCR, WB, BA	[142,156,157,158]
Hepatocellular carcinoma	↑/↓	↑ *	+/-	H, M, C	BA, MS, qPCR, WB, IHC	[142,159,160]
Colorectal cancer	↑/↓	↓ *	+/-	H, C	BA, IHC, ELISA	[142,162,163,164]
Thyroid cancer	↑/↓	NA	+/-	H, C	BA, qPCR, WB, IHC	[142,165]
Gastric cancer	↑/↓	NA	+/-	H, C	IHC	[166,167]
Prostate cancer	↑/↓	NA	-	H, M	BA, qPCR, IHC	[142,161]
Kidney cancer	↓	NA	+	H	BA	[142]

↑, upregulated expressions of PRDX6; ↓, downregulated expressions of PRDX6; +, promoting tumorigenesis; -, inhibiting tumorigenesis; NA, not applicable; H, human; R, rat; M, mouse; C, cell; F, fish (Xiphophorus); BA, bioinformatics analysis; 2-DE, two-dimensional electrophoresis; qPCR, quantitative polymerase chain reaction; WB, Western blotting; ELISA, enzyme-linked immunosorbent assay; IHC, immunohistochemistry; IF, immunofluorescence; MS, mass spectrometry; *, examined in peripheral blood serum; ^#^, examined in interstitial fluid.

**Figure 2:**
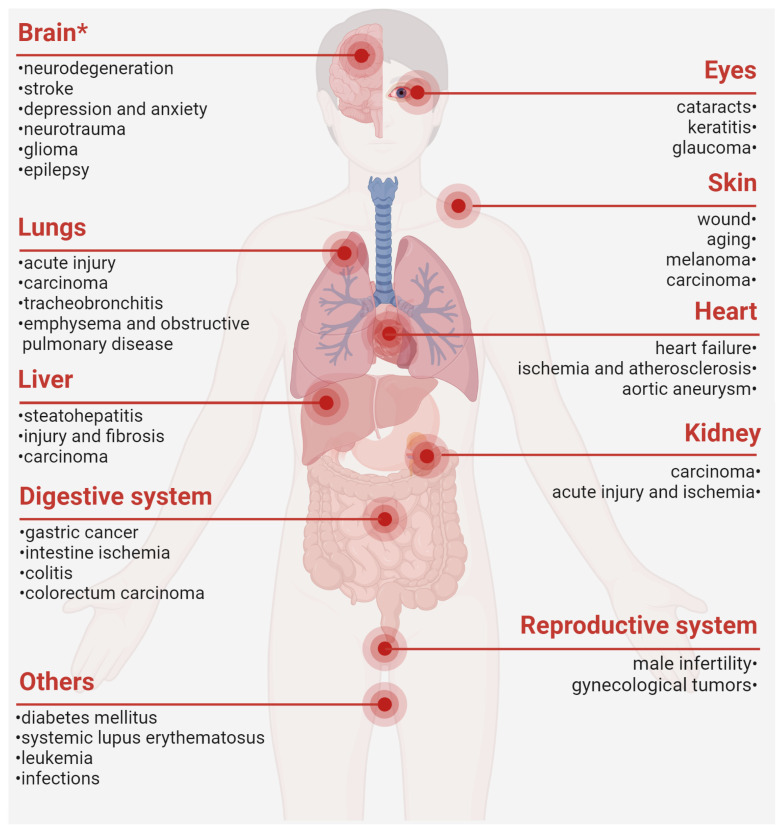
PRDX6-related disorders. *, our previously published review. The figure was created with Biorender.com [1,2,8,13,15,17,19,21,22,23,24,25,30,36,39,41,45,46,47,49,75,83,84,86,100,110,111,114,125,142,144,145,146,147,148,149,150,151,152,159,160,162,163,164,166,167,175,179,180,181,182,183,184,185].

Taken together, PRDX6 is dysregulated in a cluster of diseases and closely involved in their pathogenesis, thereby substantiating the conceptualization of PRDX6-related disorders (PRD) (Figure 2). Mechanistically, PRDX6 orchestrates its effects across diverse systems via shared molecular signaling pathways, such as MAPKs, TLR (Toll-like receptors)/NF-κB, and ferroptosis [1,73,178]. For instance, PRDX6 has been implicated in neurodegeneration [1], male infertility [8,140], and senescence and cancer [10] by attenuating oxidative stress and inflammation. However, current research may still present a conundrum regarding whether the upregulation of PRDX6 is protective, neutral, or pro-pathogenic in specific diseases. Future investigations employing well-characterized mutant or transgenic animal models may better delineate the role of PRDX6 in disease progression and remission, thereby clarifying its causal relation in disease etiology.

Moreover, PRDX6 holds promise as a diagnostic biomarker or therapeutic target, particularly for neurological and oncological diseases. Future comprehensive and large-scale studies are imperative to evaluate its specificity and sensitivity as a potential clinical indicator. Given recent recognitions about the critical role of peripheral immunity in disease pathogenesis, it is compelling to explore the origin of the PRDX6 antibody and its roles in disease onset and progression. Only when these uncertainties are resolved can we advance towards the development of PRDX6-targeted interventions.

## 5. Therapeutic Potential of Exogenous Supplementation of PRDX6

Although PRDX6 has been considered an antioxidant enzyme playing a significant role in maintaining cellular redox homeostasis, the efficacy of its exogenous administration for conferring antioxidant protection remains controversial. The challenge lies in the intracellular delivery of a mature protein because of its impermeability to the cell membrane. Previously, an HIV transactivating transduction (TAT) domain (RKKRRQRRR) has been engineered to the N-terminal region of the PRDX6, which enables 100% delivery across both the plasma membrane and blood–brain barrier. With this strategy, researchers have demonstrated that administration of PRDX6 not only restores its gene promoter activity [11] but protects against oxidative stress, apoptosis, and inflammatory insults in both the lens epithelial cells and retinal ganglion cells [90,186,187,188,189].

Under normal conditions, studies using recombinant PRDX6 labeled with fluorescein isothiocyanate (FITC) and molecular docking techniques have observed an approach of PRDX6 to the cell surface, serving as a close engagement with the membrane TLR4 receptor rather than a simple interaction [123]. Moreover, a part of the PRDX6 protein can intracellularly penetrate via its aiPLA2 activity, presumably accumulating in the transport vesicles from endoplasmic reticulum rather than in the nucleus [73,123,190]. More intriguingly, in vivo studies indicate that intravenous pretreatments with PRDX6 not only mitigate ischemia/reperfusion (I/R)-induced injuries in both intestinal and renal tissues [179,180] but also reduce mortality rates, glycemia- and cytokine-induced cytotoxicity, and radiation-induced injuries [175,191]. An intraperitoneal injection of exogenous PRDX6 protein has been shown to enhance blood–brain barrier integrity and improve overall health status in mice suffering from relapsing/remitting experimental autoimmune encephalomyelitis, which may be mediated by a reduced expression of NADPH oxidase 1/4 (NOX1/4) in the brain [192]. Other researchers have also demonstrated that topical administration or subcutaneous injection of PRDX6 can ameliorate wound-induced scar formation, ultraviolet-induced corneal injury, and myocardial ischemic damage in rat models [181,182,183].

Mechanistically, the administration of the recombinant PRDX6 protein restores cellular gene signatures associated with oxidative stress response (e.g., NRF2 and MAPKs), cell cycle regulation and apoptosis (e.g., p21, p53, and caspase-3), glucose metabolism (e.g., glucose transporter 2), cellular senescence (e.g., SA-β-Gal), and inflammatory pathways (e.g., TLR4, NF-κB, and IL-1β) [175,190,193,194]. Although the specific mechanisms by which PRDX6 triggers these gene cascades remain obscure, these findings strongly support the immense therapeutic potential of PRDX6 in attenuating oxidative stress and relevant pathological conditions, particularly in ocular disorders such as cataract and neurological diseases including encephalomyelitis.

The dual enzymatic properties of PRDX6 are essential for conferring its cytoprotective effects. Studies of site-directed mutagenesis have demonstrated that the C47 mutation, which results in a loss of Gpx activity of PRDX6, significantly attenuates its protective capacity against I/R-induced renal and intestinal injuries [179,180] and irradiation-induced lens epithelial cell damage [186,187]. However, the C47S mutant PRDX6 retains partial radioprotection against X-ray-induced oxidative stress, possibly owing to its extracellular form as a signaling molecule [123,191]. On the other hand, the S32 mutation in PRDX6, while abolishing its aiPLA2 enzymatic activity, displays greater cardioprotective effects against myocardial injury through its augmented antioxidant capacity [183]. These findings collectively reveal that the biological functions of PRDX6 are mediated through a complex interplay between its Gpx and aiPLA2 enzymatic activities, along with its extracellular penetration capability.

It is an intriguing question to inquire about the distribution of PRDX6 protein in the body upon intravenous administration. Pre-clinical studies have reported that PRDX6 is specifically localized within the blood vessel lumen after intravenous injection but can penetrate through the vascular wall [179]. Recent investigations have shown that PRDX6 exhibits a diffuse distribution in intestinal epithelium 15 min later, with approximately 50% remaining in circulation one hour after intravenous administration [179,191]. Pharmacokinetic analyses reveal that PRDX6 retains its highest concentration in the bloodstream for 10 min post-intravenous delivery but gradually decreases over time, by around 80% within the first hour and about 30% after six hours [180]. These data suggest that intravenous administration of PRDX6 attains peak concentrations in the blood and enables rapid distribution to multiple organs within 10–15 min, suggesting potential applications particularly under emergency or critical conditions.

Several critical issues remain to be addressed regarding the therapeutic application of recombinant PRDX6 protein. First, significant gaps remain in our understanding of its safety profile, therapeutic efficacy, and potential adverse effects, particularly the immunogenicity risk associated with repeated administration. Further investigations are awaited to elucidate the pharmacokinetic properties, immune responses, post-translational modifications (e.g., hyperoxidation), and comparative effectiveness in relation to established antioxidant therapies. Second, balancing the Gpx and aiPLA2 enzymatic activities within recombinant PRDX6 protein remains a significant challenge, as excessive PRDX6 activity in certain contexts may promote inflammation through arachidonic acid release. Additionally, the therapeutic window, including optimal dosage, administration timing, and delivery methods, may need to be tailored to specific disease pathophysiology. Lastly, repeated administration of recombinant PRDX6 protein may pose clinical challenges for the management of chronic diseases, including potential economic burdens. Alternative delivery strategies, such as viral vectors, mRNA-based therapies, and nanoparticle-mediated delivery systems, may overcome the limitations associated with repeated protein administration.

Despite these challenges, accumulated pre-clinical studies have demonstrated that exogenous supplementation of recombinant PRDX6 protein, regardless of the dosage or administration strategies, effectively mitigates oxidative stress-related pathological alterations (Table 4). This therapeutic approach offers distinct advantages, particularly its capacity to rapidly induce both local and systemic antioxidant and immunomodulatory effects. These findings also provide a valuable foundation for the development of novel pharmaceutical interventions targeting oxidative stress-related disorders. Nevertheless, the path to clinical translation remains complex and protracted, requiring sustained effort and extensive exploration to address these barriers.

## 6. Conclusions and Future Perspectives

PRDX6, a moonlighting protein endowed with diverse enzymatic activities, has garnered significant attention in recent years. Although it is predominantly localized within the cellular compartment, this enzyme demonstrates the capacity for extracellular secretion under specific pathophysiological conditions. The expression profile and subcellular distribution of PRDX6 are dynamically regulated by a spectrum of external stimuli, which can be systematically classified into chemical, physical, and biological categories. Furthermore, both internal and external stressors, such as ROS, pH fluctuations, and post-translational modifications, can modulate the structural dynamics and functional switches of this enzyme. Given these findings, it is interesting to speculate that PRDX6 may play a pivotal role not only in intercellular signaling transduction, but also in mediating gene-environmental interactions. The inherent susceptibility of PRDX6 to various stressors qualifies it as a promising biomarker for disease recognition and treatment monitoring.

Accumulating evidence has indicated the dysregulation and pathophysiological involvement of PRDX6 in various disease states, particularly in neuropsychiatric disorders and oncological conditions. Emerging genetic studies have revealed significant associations between the PRDX6 gene polymorphisms and individual susceptibility to systemic lupus erythematosus [184] and chronic obstructive pulmonary disease [185], emphasizing its critical roles in disease-related genetic variability. Whilst the precise mechanisms need further elaboration, the dysregulation of PRDX6 appears to indicate a common molecular pathway or pathological event in multiple diseases. Based on these scientific observations, we propose the concept of PRDX6-related disorders (PRD), which refers to a spectrum of diseases mediated by or associated with dysregulation of PRDX6 expression or enzymatic activity.

The remarkable antioxidant capacity of PRDX6 has prompted investigations into its preventive and therapeutic potential through exogenous supplementation, either as the native protein or in compound formulations, particularly for conditions involving oxidative stress, a ubiquitous pathological process across numerous diseases. More intriguingly, emerging evidence suggests that PRDX6 may play a critical role in immune modulation, given its presence in various immune cells (e.g., neutrophils, monocytes, and macrophages) and its detection in bodily fluids alongside a specific antibody. These findings suggest new avenues for the development of PRDX6-targeted immunotherapeutic strategies for PRD management.

The present comprehensive review systemically examined the various stressors regulating the PRDX6 expression and its structure-based functional switches, introduced the novel concept of PRDX6-related disorders, and evaluated the therapeutic potential of exogenous PRDX6 administration in oxidative stress-related pathologies. Our primary objective was to provide a conceptual framework for and offer a macroscopic perspective into the response of PRDX6 to diverse stressors, rather than delving into intricate mechanistical investigations. While we acknowledge certain limitations in this approach, we have also incorporated discussions of several key stressor-induced regulatory mechanisms for the PRDX6 expression and activity. Collectively, this review positions PRDX6 as a cellular orchestrator in response to both internal and external stressors, underscoring its clinical potential in disease diagnosis and therapeutic intervention.

## Figures and Tables

**Table 1:** In vivo regulations of PRDX6 induced by external stimuli.

Species	Samples	External Stimuli	Alteration of PRDX6	Upstream Molecules	References
Human	Lung	Sulfur mustard	↑	N/A	[14]
	Blood	High-intensity interval training	↑	N/A	[34]
		Liraglutide and metformin	↓	GLP-1R	[40]
	Sperm	Date palm pollen	↑	N/A	[23]
		4-tert-octylphenol	↓	cAMP-PKA/C	[21]
Rat	Lung	SiO_2_	↑	SP-A	[41]
	Testis	Di-n-butyl phthalate; Curcumin	↑	SP1	[24,42]
	Epididymis	Tert-butyl hydroperoxide	↑	N/A	[25]
	Placenta	MgSO_4_	↑	NRF2	[12]
	Bile duct	Thioacetamide	↑	Wnt7a/b	[43]
	Pancreas	Hydroxytyrosol	↑	N/A	[44]
	Heart	Adriamycin	↓	N/A	[45]
	Colon	Voluntary exercise; Hydrogen sulfide	↑	N/A	[15,36]
		2,4,6-trinitrobenzenesulfonic acid	↓	N/A	[15]
	Liver	High-fat diet; whole grain	↑	N/A	[46]
		CCl_4_	↑	N/A	[19]
		Chronic ethanol exposure	↓	N/A	[17]
	Skin	Aloe vera	↑	N/A	[47]
		Electron beam (45 Gy)	↓	miR-214	[30]
	Brain	Ferrostatin-1	↑	Fer1	[48]
Mouse	Blood	Angiotensin II	↑	N/A	[49]
	Brain; lung	Hyperbaric oxygen exposure	↑	N/A	[33]
	Pancreas; Salivary glands	Epigallocatechin-3-gallate	↑	p38 and JNK	[50]
	Cochleae	All-trans retinoic acid	↑	RARα	[51]
		CoCl2	↑	HIF-1α and NRF2	[13]
	Spleen	Ionizing radiation (10 Gy)	↑	N/A	[27]
	Kidney	Andrographolide sodium bisulfite	↑	Mitochondrial complex I	[52]
		NH_4_Cl	↑	AE1	[53]
	Testis	Melatonin	↑	N/A	[54]
		Di-2-ethylhexyl phthalate	↓	N/A	[55]
		Paclitaxel	↓	SIRT1 and NRF2	[22]
	White adipose	Buthionine sulfoximine	↑	NRF2	[16]
	Skin	Puerariae lobatae radix	↑	REV-ERBα/BMAL1/NRF2	[29]
		Ultraviolet-B	↓		
	Brain	Lead	↓	N/A	[56]
	Lung	Snake venom toxin	↓	AP-1	[57]
		Paraquat; Lipopolysaccharide; Ricin; Thiacremonone; H1N1 influenza virus	↓	N/A	[37,58,59,60]
	Liver	Salvia miltiorrhiza polysaccharide	↑	N/A	[38]
		Clonorchis sinensis	↑	NF-κB; KEAP1/NRF2, HIF-1α, and C/EBPβ	[39]
		Single-walled carbon nanotubes; Proton irradiation (2 Gy); Ionizing radiation (10 Gy)	↑	N/A	[26,27,28]
		Chronic ethanol treatment	↓	NF-κB; MEK1/2	[18]
		CCl_4_	↓	N/A	[20]
	Colon	CaCO_3_	↑	FoxM1 and NF-κB	[61]
		Liquiritin	↑	Direct binding	[62]
		Dextran sulfate sodium	↓	N/A	[62]
Rabbit	Oviduct	Mating and fertilization	↑	N/A	[35]
	Liver	Olmesartan	↓	AT1	[63]
Bovine	Ovary	Melatonin	↑	MTNR1A/B	[64]
		α-pinene	↑	NRF2	[65]
		Aloe vera	↑	N/A	[66]
		Thymol	↓	N/A	[67]
Fish	Gills	Citrate	↑	N/A	[68]
	Liver	Soybean oil; low temperature	↑	N/A	[31,69]
		High temperature and hypoxia	↓	N/A	[32]

↑, upregulated expressions of PRDX6; ↓, downregulated expressions of PRDX6; N/A, not applicable; GLP-1R, glucagon-like peptide-1 receptor; cAMP-PKA/C, cyclic adenosine monophosphate-protein kinase A/C; SP-A, surfactant protein A; SP1, specificity protein 1; NRF2, nuclear factor erythroid 2-related factor 2; NF-κB, nuclear factor kappa B; Fer1, ferrostatin-1; JNK, c-Jun N-terminal kinases; RARα, retinoic acid receptor alpha; HIF-1α, hypoxia-inducible factor 1 alpha; AE1, anion exchanger 1; SIRT1, sirtuin 1; REV-ERBα, also known as NR1D1, nuclear receptor subfamily 1 group D member 1; AP-1, activation protein 1; BMAL1, brain and muscle ARNT-like protein 1; KEAP1, Kelch-like ECH-associated protein 1; C/EBPβ, CCAAT/enhancer-binding protein beta; MEK1/2, MAPK (mitogen-activated protein kinase)/ERK (extracellular-signal-regulated kinase) kinase 1/2; FoxM1, Forkhead box M1; AT1, angiotensin receptor; MTNR1A/B, melatonin receptors types 1A/B.

**Table 2:** In vitro regulations of PRDX6 induced by external stimuli.

Species	Various Cell Lines		External Stimuli	Alterations of PRDX6	Upstream Molecule	References
Human	Colorectal epithelial cell	Caco-2	Roasted coffee extracts	↑	ARE	[93]
		SW-480 SW-620	5-fluorouracil	↑	N/A	[94]
		DLD-1	Baicalein	↑	N/A	[80]
		HT-29	Portoamides	↑	N/A	[95]
		HCT-8	Curcumin (25 µM, for 72 h)	↓	N/A	[77]
	Alveolar epithelial cell	H460	Ionizing radiation (2 Gy)	↑	TRIAP1	[70]
		HCC-827	Gefitinib	↑	EGFR	[96]
		A549	Ionizing radiation (2 Gy)	↑	TRIAP1	[70]
			Keratinocyte growth factor	↑	NRF2/ARE	[97]
			Dexamethasone (1 µM)	↑	GRE	[97]
			Endothelial growth factor	↑	EGFR/PI3K	[98]
			Curcumin (109 µM, for 2 h) *	↑	N/A	[74]
			Snake venom toxin;	↓	AP-1	[57]
			Thiacremonone	↓	Direct binding	[60]
	Breast epithelial cell	MCF-7; MCF-10A	Oleuropein	↑	N/A	[99]
	Lens epithelial cell	hLEC	Curcumin (5 µM, for 48 h) Ginkgolic acid	↑	SP1	[75,100]
			Sulforaphane	↑ (6 µM)	NRF2/ARE	[101]
				↓ (>6 µM)	NRF2/KLF9	[102]
			Betulinic acid	↓	SP1	[100]
	Monocyte	THP-1	Electromagnetic fields	↑	N/A	[72]
		U937	Cigarette smoke condensate; Ethanol; Darunavir/ritonavir	↓	N/A	[103,104]
		U1	Cigarette smoke condensate	↓	N/A	[103]
	Hepatocyte	Huh-7	Luteolin	↑	N/A	[81]
			(E)-4-chloro-2-((3-ethoxy-2-hydroxybenzylidene)amino)phenol	↓	p38β/SAPK	[105]
		LMH	Andrographolide	↑	N/A	[82]
		HepG2	Curcumin (1737 µM, for 72 h) *	↓	p53	[78]
			Hesperidin	↓	N/A	[106]
	Vascular cell	UVEC	Sheer stress	↑	KLF2/miR-27b/CSE	[107]
			Angiotensin II	↓	AT1R	[108]
	Retinal epithelial cell	RPE-1	Ionizing radiation (20 Gy); BrdU	↑	N/A	[71]
		APRE-19	H_2_O_2_ (300, 400, or 500 µM, for 6 h; 500 or 1000 µM, for 48 h); Blue light	↓	N/A	[84,86]
	Salivary gland epithelial cell	HSG	Epigallocatechin-3-gallate	↑	p38 and JNK	[50]
			H_2_O_2_ (100 µM, for 30 min)	↓	N/A	[50]
	Astrocyte	A172 HA-sp	H_2_O_2_ (50, 100, or 200 µM, for 24 h)	↑	N/A	[76]
			Curcumin (1 µM, for 24 h)	↓	N/A	[76]
	Neuron	SH-SY5Y	Hyperglycemia + bupivacaine	↓	KLF9	[109]
	Trabecular meshwork cell	TM	H_2_O_2_ (100 µM, for 8 h)	↓	N/A	[85]
	Bronchial epithelial cell	BEAS-2B	Diesel exhaust particles; Cigarette smoke extract	↓	N/A	[110,111]
		16-HBE	Cigarette + LPS (1 µg/mL, for 8 h)	↓	N/A	[112]
	Oral epithelial cell	HN13	Tert-butyl hydroperoxide	↑ (50 µM), ↓ (250 µM)	N/A	[113]
	Leukemia cell	K562	Andrographolide-lipoic acid	↑	N/A	[83]
		Jurkat	Alkaline serine protease	↓	N/A	[114]
	Fibrosarcoma cell	HT-1080	Korean Scutellaria baicalensis flavonoids	↓	N/A	[115]
	Embryonic kidney cell	HEK-293	TNFα (500–2500 U/mL, for 5, 7, or 9 h) *	↑	TNFα/NOX1	[88]
	Renal tubular epithelial cell	HK-2	High glucose	↓	N/A	[116]
	Embryonic lung fibroblast	MRC-5	Ionizing radiation (20 Gy); BrdU	↑	N/A	[71]
	Ovarian cell	SKOV-3	Cisplatin (40 µM)	↑ (3–9 h), ↓ (15–24 h)	N/A	[117]
	Esophageal epithelial cell	Kyse510	Dioscin	↓	N/A	[118]
	Melanocytes	A375	Endothelial growth factor	↑	EGFR	[98]
Rat	Cerebellar granule neuron	CGN	Persistent organic pollutants	↑	N/A	[119]
	Sertoli cell	TSC	Nonylphenol	↑	N/A	[120]
	Alveolar epithelial cell	AT-II	Keratinocyte growth factor	↑	NRF2/ARE	[97]
	Condylar chondrocyte	RCC	Glycyrrhizin	↑	NRF2	[79]
			H_2_O_2_ (100 µM, for 24 h)	↓	N/A	[79]
	Lens epithelial cell	rLEC	Sulforaphane	↑ (6 µM)	NRF2/ARE	[101]
				↓ (>6 µM)	NRF2/KLF9	[102]
	Pancreatic beta cell	RINm5F	TNFα (1850 U/mL, for 24 h); IFNγ (140 U/mL, for 24 h); IL-4	↓	TNFα; IFNγ; IL-4	[89]
	Retinal ganglion cell	RGC-5	TNFα (50ng/mL, for 10 days; 100 ng/mL, for 72 h)	↓	TNFα	[90]
			Glutamate (5 mM)	↓	NMDAR	[90]
Mouse	Lens epithelial cell	mLEC	Ginkgolic acid	↑	SP1	[100]
			Betulinic acid	↓	SP1	[100]
	Hepatocyte	Hepa1-6	Mithramycin A	↓	SP1	[121]
		H2.35	Dexamethasone (0.1 µM)	↑	N/A	[87]
			Keratinocyte growth factor	↑	PKC/MAPK	[87]
			TNFα (10 ng/mL, for 8 or 24 h)	↑	TNFα/PKC/MAPK	[87]
			BAY117082	↑	NF-κB	[87]
			H_2_O_2_ (1000 µM, for 8 or 24 h)	↑	N/A	[87]
	Auditory cell	HEI-OC1	All-trans retinoic acid	↑	RARα	[51]
			CoCl_2_	↑	HIF-1α and NRF2	[13]
			H_2_O_2_ (25 µM, for 9 h)	↑	N/A	[13]
			H_2_O_2_ (100 µM, for 24 h)	↓	N/A	[13]
	Glomerular podocyte	MPC5	High glucose	↓	SP1	[122]
	Tracheobronchial epithelial cell	TBE	LPS (1, 10, or 20 µg/mL, for 8 or 24 h)	↓	N/A	[91]
	Embryonic fibroblast cell	3T3	X-ray (16 Gy)	↑	N/A	[123]
	Macrophage	BMM	NO	↑	NO/Srx	[92]
			IFNγ (100 U/mL) + LPS (0.5 µg/mL), for 18 h	↑	N/A	[92]
Porcine	Granulosa cell	GCs	Gossypol	↑	N/A	[124]
	Kidney cell	PK-15	Foot-and-mouth virus	↓	N/A	[125]

↑, upregulated expressions of PRDX6; ↓, downregulated expressions of PRDX6; ARE, antioxidant response element in DNA; TRIAP1, TP53-regulated inhibitor of apoptosis 1; EGFR, epidermal growth factor receptor; NRF2, nuclear factor erythroid 2-related factor 2; GRE, glucocorticoid response element in DNA; PI3K, phosphatidylinositol 3-kinase; AP-1, activation protein 1; SP1, specificity protein 1; KLF9, Kruppel-like factor 9; SAPK, stress-activated protein kinase; CSE, cystathionine gamma lyase; AT1R, angiotensin II type 1 receptor; BrdU, a senescence inducer; TNFα, tumor necrosis factor alpha; NOX1, NADPH oxidase 1; IFNγ, interferon gamma; IL-4, interleukin 4; NMDAR, N-methyl-D-aspartate receptor; PKC, Protein kinase C; MAPK, mitogen-activated protein kinases; BAY117082, an inhibitor of IκB/IKK; NF-κB, nuclear factor kappa B; NO, nitric oxide; LPS, lipopolysaccharide; Srx, sulfiredoxin; Gy, Gray. *, indicating a conversion of the concentration unit (curcumin: M = 368.38, referred to from the Sigma website; TNFα: ED_50_ [1 unit (U)] = 20–100 pg/mL, referred to from the R&D Systems website).

**Table 4:** Protective effects of exogenous PRDX6 supplementation.

Study	Model	Dose	Strategies	Significance	References
In vivo	SCR rat	20 μg	Every 72 h, for 2 weeks (TAT, s.c.i.)	Delay the progression of cataracts	[186]
	UCI rat	30 μg	4 times per day, for 14 days after injury (t.a.)	Suppress ultraviolet radiation-induced inflammation and neovascularization	[182]
	IW rat	0.5 mg/mL	1 and 3 h after incision, 2 times per day (t.a.)	Accelerate incised wound healing and decrease the size of the scar formation	[181]
	MII rat	2 μg/kg	for 14 days (s.i.)	A certain repair effect on myocardial injury	[183]
	ASMAO rat	10 μg/g	15 min before I/R injury (i.v.)	Protect against I/R-induced damage of small intestine	[179]
	T1DM mouse	20 μg/g	The first day and repeatedly on the eighth day (i.v.)	Decrease the mortality rate and glycemia, lower splenocytic apoptosis and plasma cytokines, and increase the pancreatic β cell mass	[175]
	RIRI mouse	20 μg/g	15 min before I/R injury (i.v.)	Reduce the degree of kidney I/R injury	[180]
	Irradiated mouse	20 μg/g	15 min before X-ray irradiation (i.v. or i.p.)	Reduce the radiation-induced organism injuries (e.g., leuko- and thrombopenia, small intestine, and red bone marrow)	[191]
	EAE mouse	6 μg/g	Days 2, 7, and 10 after EAE induction (i.p.)	Improve the EAE-induced symptoms and blood–brain barrier dysfunction	[192]
In vitro	hLECs	10 and 20 μg/mL	Pretreated before ultraviolet-B irradiation (TAT)	Protect the crystalline lens cells from oxidative stress induced by ultraviolet-B irradiation	[187]
	hMSCs	10 ng/mL	Incubation for 7 days (d.a.)	Promote cell differentiation into insulin-producing cells	[193]
	RIN-m5F	150 μg/mL	30 min before additions of cytokines (d.a.)	Reduce the inflammation cascades, cytokine-induced cytotoxicity and apoptosis, and oxidative stress	[175]
	rRGCs	4 μg/mL	3 h before additions of glutamate and/or TNFα (TAT)	Attenuate TNFα- and glutamate-induced cell death by limiting ROS levels and maintaining Ca^2+^ homeostasis	[90]
	H9C2	0.1 μg/mL	Pretreatment for 2 h (d.a.)	Attenuate isoprenaline-induced cell death, apoptosis and oxidative stress	[183]
	r/mLECs	5–10 μg/mL	24 h before additions of H_2_O_2_ and/or TGF1β (TAT)	Protect against H_2_O_2_- and TGF1β-induced cell death and oxidative stress, and inhibit ROS-mediated adverse signaling	[186]
	mLECs	4 μg/mL	Incubation for 72 h (TAT)	Restore the Prdx6 gene promoter activity by attenuating SMAD3/TGF1β signaling	[11]
		10 μg/mL	Incubation for 96 h (TAT)	Attenuate adverse signaling in cells and maintain cellular homeostasis	[188]
		N/A	Pretreated before hypoxia stress (TAT)	Optimize hypoxia-induced overstimulation of endoplasmic reticulum stress	[189]
	Raw 264.7	150 μg/mL	Within the first hour after LPS (d.a.)	Decrease lipolyaccharide-induced ROS, apoptosis, and pro-inflammation	[194]
	3T3	300 μg/mL	Incubation for 3 h; 4 h after irradiation (d.a.)	Increase survival, suppress oxidative stress, senescence, apoptosis, and necrosis under X-ray exposure	[123,190]

SCR, Shumiya cataract rat model of hereditary cataract; TAT, human immunodeficiency virus (HIV)-TAT domain (RKKRRQRRR) used to construct a recombinant TAT-linked PRDX6 protein; s.c.i., subconjunctival injection; UCI, ultraviolet-induced corneal injury rat; t.a., topical administration; IW, incision would rat; MII, myocardial ischemia injury rat model induced by isoprenaline; s.i., subcutaneous injection; ASMAO, acute superior mesenteric artery occlusion rat; I/R, ischemia–reperfusion injury; i.v., intravenous injection (vena caudalis); T1DM, type 1 diabetes mellitus induced by alloxan; RIRI, renal ischemia–reperfusion injury mouse; i.p., intraperitoneal injection; EAE, experimental autoimmune encephalomyelitis; hLECs, human lens epithelial cells; hMSCs, human mesenchymal stem cells; d.a., direct administration; RIN-m5F, rat insulinoma m5F β cells; rRGC, rat retinal ganglion cells; TNFα, tumor necrosis factor alpha; ROS, reactive oxygen species; H9C2, rat embryonic myocardial cells; r/mLECs, rat or mouse lens epithelial cells; TGF1β, transforming growth factor 1 beta; SMAD3, SMAD family member 3; Raw 264.7, murine macrophage-like cells; 3T3, mouse embryonic fibroblast cells.

## Abbreviations

PRDXs	peroxiredoxins
Cys	cysteine
-SH	reduced state
-SOH	sulfenic state
-SO_2_H	sulfinic acid
-SO_3_H	sulfonic acid
ATP	adenosine triphosphate
Gpx	glutathinone peroxidase
aiPLA2	acidic calcium-independent phospholipase
LPCAT	lysophosphatidylcholine acyl transferase
SP1	specificity protein 1
NRF2	nuclear factor erythroid 2-related factor 2
HIF-1α	hypoxia-inducible factor 1 alpha
MgSO_4_	magnesium sulfate
CoCl_2_	cobalt chloride
NF-κB	nuclear factor kappa B
MAPKs	mitogen-activated protein kinases
CCl_4_	carbon tetrachloride
MANF	mesencephalic astrocyte-derived neurotrophic factor
PKA/C	protein kinase A/C
SIRT1	sirtuin 1
BMAL1	brain and muscle ARNT-like protein 1
C/EBPβ	CCAAT/enhancer-binding protein beta
H_2_O_2_	hydrogen peroxide
IL-1β	Interleukin 1β
PI3K	phosphatidylinositol 3-kinase
Akt	protein kinase B
TNFα	tumor necrosis factor alpha
GLP-1R	glucagon-like peptide-1 receptor
EGFR	epidermal growth factor receptor
NMDAR	N-methyl-D-aspartate receptor
ROS	reactive oxygen species
PRD	PRDX6-related disorder
TLR	Toll-like receptor
TAT	HIV transactivating transduction
FITC	fluorescein isothiocyanate
I/R	ischemia/reperfusion
NOX1/4	NADPH oxidase 1/4
